# Uroprotective and pain-relieving effect of dietary supplementation with micronized palmitoyl-glucosamine and hesperidin in a chronic model of cyclophosphamide-induced cystitis

**DOI:** 10.3389/fvets.2023.1327102

**Published:** 2024-01-04

**Authors:** Enrico Gugliandolo, Gianluca Antonio Franco, Ylenia Marino, Alessio Filippo Peritore, Daniela Impellizzeri, Marika Cordaro, Rosalba Siracusa, Roberta Fusco, Ramona D’Amico, Francesco Macrì, Rosanna Di Paola, Salvatore Cuzzocrea, Rosalia Crupi

**Affiliations:** ^1^Department of Veterinary Science, University of Messina, Messina, Italy; ^2^Department of Chemical, Biological, Pharmaceutical and Environmental Science, University of Messina, Messina, Italy; ^3^BioMorf Department, University of Messina, Messina, Italy; ^4^Department of Pharmacological and Physiological Science, Saint Louis University School of Medicine, Saint Louis, MO, United States

**Keywords:** cystitis, palmitoyl-glucosamine, hesperidin, mast cells, visceral pain, urothelium, neuroinflammation, ALIAmides

## Abstract

**Introduction:**

Feline idiopathic cystitis is a common, chronic-relapsing disorder of the lower urinary tract. In addition to environmental modification/enrichment, long-term and safe treatment targeting specific pathophysiological changes may be of help. In this context, effective dietary interventions hold clinical promise. Palmitoyl-glucosamine (PGA) and hesperidin (HSP) are safe and authorized feed ingredients for animal nutrition under European regulations.

**Methods:**

The current study aimed to investigate whether a 3:1 mixture of micronized PGA and HSP could represent a novel mechanism-oriented approach to chronic cystitis management. A newly validated rat model of cyclophosphamide (CYP)-induced chronic cystitis was used (40 mg/kg, three intraperitoneal injections every 3rd day). Animals were randomized to orally receive either vehicle or PGA-HSP at a low (72 + 24 mg/kg) or high (doubled) dose for 13 days, starting 3 days before the chronic CYP protocol, with mesna (2-mercaptoethane-sulfonate) being used as a reference drug.

**Results:**

Higher PGA-HSP dose was effective at relieving chronic visceral pain, as measured by mechanical allodynia test (von Frey test). The severity of cystitis was also significantly improved, as shown by the reduced sonographic thickening of the bladder wall, as well as the decrease in edema, bleeding and bladder to body weight ratio compared to the vehicle treated group. A significant decrease of MPO activity, MDA level and fibrosis at Masson’s trichrome staining was also observed in animals administered PGA-HSP in comparison to vehicle treated ones. The CYP-induced increase in bladder mRNA expression of pro-inflammatory cytokines was also significantly counteracted by the study mixture. Moreover, CYP-induced bladder mast cell accumulation and releasability were significantly decreased by PGA-HSP (even at the low dose), as determined by metachromatic staining, chymase and tryptase immunostaining as well as enzyme-linked immunosorbent assay for histamine and 5-hydoxytriptamine.

**Discussion:**

PGA-HSP is able to block CYP-induced decrease of tight junction proteins, claudin-1 and occludin, thus preserving the urothelial bladder function. Finally, neuroinflammatory changes were investigated, showing that dietary supplementation with PGA-HSP prevented the activation of neurons and non-neuronal cells (i.e., microglia, astrocytes and mast cells) at the spinal level, and counteracted CYP-induced increase of spinal mRNA encoding for pro-inflammatory cytokines. Altogether, the present findings confirm the uroprotective and pain-relieving effect of PGA-HSP and pave the way to potential and relevant clinical applications of the study supplement in feline idiopathic cystitis.

## 1 Introduction

Feline idiopathic cystitis (FIC) is a naturally occurring bladder disease with recurring or chronic persistent episodes, characterized by bladder inflammation and pain (1–4). Notably, it is frequently considered the preferred model for human interstitial cystitis/bladder pain syndrome (IC/BPS), a debilitating chronic pain syndrome which indeed shares many histologic and clinical features with FIC (4, 5). Clinically, both FIC and IC/BPS have unknown etiology and their respective diagnosis is made on exclusion of other causes. The two diseases are both characterized by lower urinary tract symptoms, such as urinary frequency and painful urination. On the histology side, increased number of mast cells, accumulation of leukocytes, bladder vascularization changes and impaired urothelium barrier function have been reported in human as well as feline patients. Oxidative damage is a further, albeit less investigated, common feature (6, 7) so much so that promoting antioxidant responses proved to be a protective strategy in chronic cystitis (8, 9). Sensitization of bladder afferents and alterations in the spinal cord circuits responsible for bladder sensation have also been repeatedly considered to play a role in cystitis-associated chronic visceral pain (10, 11).

The primary treatment objective of an acute episode is to provide pain relief. Pharmacotherapy of both FIC and IC/BPS includes oral anti-inflammatory and/or analgesic drugs and intravesical instillation (e.g., hyaluronic acid, dimethyl sulfoxide) (3, 12–14). To increase “disease-free time” in patients with recurrent idiopathic cystitis is a further treatment goal. In this context, dietary interventions, therapeutic urinary diets and environmental enrichment have shown some promising results in FIC (3, 15), although the waxing/waning nature of clinical signs makes it difficult to properly evaluate benefits of potential treatment options. Overall, a number of different approaches have been developed in order to address either the intra-or inter-episode needs (or hopefully both), but a long-lasting and safe management is still far from being satisfactory. Recently, a rat model of cyclophosphamide (CYP)-induced chronic cystitis has been validated and shown to share strong similarities with IC/BPS (and its feline counterpart), like the development of persistent inflammatory response, painful behavior, bladder edema and urothelial damage (16). This provides a unique opportunity to investigate new potential approaches, discover mechanisms underlying the effects, and identify cellular and molecular targets. Palmitoyl-glucosamine (PGA) is a natural monosaccharide-based glycolipid (17) and a member of the prohomeostatic ALIAmide family, i.e., a group of naturally occurring lipids acting through the so-called autacoid local injury antagonism (ALIA) mechanism (18). Originally hypothesized by the Nobel Prize winner Rita Levi Montalcini (19), the mechanism mainly consists in the down-modulation of hyperactive immune-inflammatory cells, especially mast cells, for protective purposes (20). The ALIAmide parent molecule palmitoyl-ethanolamide (PEA) reduced referred hyperalgesia in turpentine-induced inflammation of the urinary bladder (21), counteracted nerve growth factor-induced reduction in micturition threshold (22) and exerted a protective role in CYP-induced acute cystitis (23). Moreover, when co-micronized with the antioxidant polydatin (24), PEA decreased pain severity and urinary frequency in IC/BPS patients (25). Few data are currently available on PGA and its effect on cystitis has not been investigated yet. Nevertheless, PGA has shown promising effects on experimental inflammation and pain models (17, 26, 27), moreover the limitations of bioavailability are significantly improved by micronization (26). A multiple mechanisms of action for PGA, have been suggested to explain these effects, among which the down-modulation of mast cells (28) and toll-like receptor 4 (TLR4) antagonism play key role (17, 27). Furthermore, PGA is supposed to act as a source of glucosamine (26), which is an interesting feature in view of the purported benefits of glucosamine administration in cystitis (29–31). A 3:1 mixture of micronized PGA and the citrus-derived antioxidant flavonoid hesperidin (HSP) has recently been formulated and hereafter referred to as PGA-HSP. Both PGA and HSP from *citrus aurantium* are safe (26, 32) and authorized for animal nutrition by the European Commission as a feed material and a feed additive, respectively (33, 34). However, to the best of our knowledge, neither were ever investigated in chronic cystitis. Given the above, the current study was designed to investigate whether dietary supplementation with PGA-HSP could afford protection against the development of CYP-induced chronic cystitis model and the associated neuroinflammatory changes at the spinal level. Mesna (2-mercaptoethane-sulfonate), a thiol compound known to reduce the risk of bladder toxicity from CYP, was used as a reference drug (35).

## 2 Materials and methods

### 2.1 Animals

This study was performed on seven-week-old female Sprague–Dawley rats (200–230 g body weight) from Envigo RMS Srl (San Pietro al Natisone, Udine, Italy). Food and water were available *ad libitum*. The University of Messina Review Board for animal care (OPBA) approved the study. Animal care was in accordance with Italian regulations on protection of animals used for experimental and other scientific purposes (D.M.116192) as well as with EEC regulations (O.J. of E.C. L 358/1 12/18/1986), and in compliance with the requirements of the Italian Legislative Decree no. 26/2014 and subsequent guidelines issued by the Italian Ministry of Health on March 16, 2015. All animal experiments complied with the EU regulations (EU Directive 2010/63) and ARRIVE guidelines.

### 2.2 Induction of cystitis

**Figure 1:**
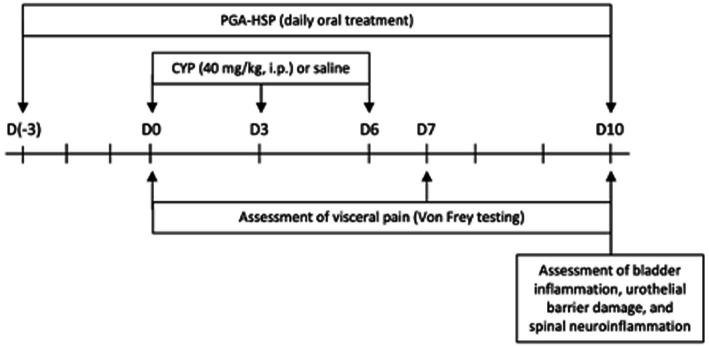
Timeline of the experimental protocol.

Given the nature of chronic FIC pathology that can afflict cats, we chose a validated chronic model of CYP-induced cystitis as shown above (16). Briefly, cyclophosphamide (CYP, 40 mg/kg) (Endoxan Baxter. Roma, Italy) was injected in the rat peritoneal cavity every 3 days (i.e., on days 0, 3 and 6) as shown in Figure 1. All rats were sacrificed by cervical dislocation at the end of the experimental protocol. A midline ventral abdominal incision was made to collect the bladder. Next, the Lumbar6-Sacral1 (L6-S1) area of the spinal cord was collected through a longitudinal incision along the midline of the back.

### 2.3 Treatment groups

The animals were randomly allocated into the following groups, sample size was calculated by G-Power (n = 6 each):

- Control: animals received i.p. injections of physiological saline (0.9% sodium chloride, Baxter, Roma, Italy) under the same experimental condition of CYP group.
- CYP: animals received three doses of CYP (40 mg/kg i.p.), as detailed in the previous paragraph and were treated with vehicle (saline).
- CYP + mesna (positive control group): animals were orally treated with the reference compound 2-mercaptoethane sodium sulfonate (Mesna, Uromixetan, Baxter. Roma, Italy), 30 min before (80 mg/kg) and 2 h after each CYP injection (160 mg/kg), as previously described (35).
- CYP + low dose PGA-HSP: animals were orally treated with PGA-HSP (72 + 24) mg/kg every 24 h, starting 3 days before the first CYP injection and until the last day of experimental protocol (day 10).
- CYP + high dose PGA-HSP: the same as the previous group, but with a double dose PGA-HSP, i.e., (144 + 48) mg/kg.

All the oral treatments were administered by oral gavage. The study supplement is a 3:1 mixture of micronized PGA and HSP.

### 2.4 Nociceptive response to mechanical stimulation

Nociceptive response was evaluated through an electronic von Frey system (Bioseb, Vitrolles, France), as previously described (16). Briefly, von Frey filaments of increasing forces were applied to previously shaved abdomen, close to the urinary bladder, before CYP or saline injection (basal, day 0), at day 7 and day 10 (Figure 1). The stimulation was applied three times at each timepoint, with the mean value being considered the mechanical threshold (expressed in grams), corresponding to the pressure that induced a behavioral response (i.e., retraction/licking of the lower abdomen, or jumping).

### 2.5 *In situ* ultrasound imaging

On day 10, under light anesthesia (3% sevoflurane in air) the bladder was non-invasively observed using an ultrasound imaging system (Esaote My Lab Vet 30 Gold, Genoa, Italy) equipped with an 18 MHz linear probe. The bladder thickness was measured on the acquired images with ImageJ software (National Institutes of Health, United States).

### 2.6 Macroscopic analyzes

On day 10, rats were euthanized with anesthetic overdose (5% sevoflurane in air) and bladder and spinal cord (L6-S1) collected for further analysis. Wet weight of each bladder was recorded. The bladders were photographed, and examined macroscopically for bleeding and edema. Bladder damage was scored according to a 4-point scale, as previously reported (36): 0-normal bladder, 1-mild (little edema and no bleeding), 2-moderate (fluid limited to the internal mucosa and little bleeding), and 3-severe (fluid in the bladder wall and internal mucosa, with evident bleeding).

### 2.7 Histological analyzes

Bladder tissues were fixed for 24 h in 4% paraformaldehyde/0.1 M phosphate-buffered saline at room temperature, dehydrated through a graded series of ethanol and embedded in Paraplast (Bio-Optica Milan, Italy). Tissue sections (7 μm) were deparaffinized with xylene, stained with hematoxylin and eosin (H&E) and evaluated by light microscopy. The degree of bladder inflammation was scored on a 4-grade scale based on submucosa edema, epithelial thinning, petechial hemorrhage, cell infiltration, and desquamation of the uroepithelium, as reported by Zhang and collaborators (37). Briefly, Grade 1 = normal control, Grade 2 = simple edema, Grade 3 = edema combined with epithelial layer cleavage and thinning, with mucosal abrasion and polymorphonuclear leucocyte (PMN) infiltration (i.e., mild cystitis) and Grade 4 = complete cystitis, i.e., increased severity and spread of all the above signs plus petechial hemorrhage (37). Bladder sections (7 μm) were also stained by Masson’s Trichrome method for collagen detection according to manufacturer protocol (Bio-Optica, Milan, Italy), and observed under light microscopy. The degree of fibrosis was evaluated as % fibrotic area (blue staining) and quantified using image analysis ImageJ software. For mast cell count, the bladder and spinal cord sections were stained with toluidine blue (Bio-Optica, Milan, Italy) and mast cells were counted in 10 cross-sections at 40x magnification in the most infiltrated area. All histological evaluations were performed by two independent pathologists blinded to the grouping. All observations were performed on Leica DM6 microscope (Leica Microsystems SpA, Milan, Italy) associated with Leica LAS X Navigator software (Leica Microsystems SpA, Milan, Italy).

### 2.8 Estimation of inflammatory infiltrate and oxidative stress

Myeloperoxidase (MPO) enzymatic activity, an index of neutrophilic granulocyte infiltration, was determined spectrophotometrically in the bladder tissues as previously described (26). MPO activity was defined as the quantity of enzyme degrading 1 μm of peroxide per minute at 37° C and was expressed in units per gram of wet tissue. Malondialdehyde (MDA) is a stable end product of lipid peroxidation and a marker of oxidative stress. MDA levels in the bladder samples were determined spectrophotometrically at 532 nm by Thiobarbituric Acid Assayas previously described (38), the levels of MDA were determined using a microplate reader at 532 nm and expressed as nmol/mg of tissue.

### 2.9 Immunohistochemical analyzes

Immunohistochemical analyzes were performed as previously described (39). Briefly the sections were incubated with the following primary antibodies: anti-occludin (1:100, WH0004950M1), anti-claudin-1 (1:100, Novus Biologicals, NBP177036), anti-mast cell chymase (CC1) (1:50, SCB sc-59586), and anti-mast cell tryptase (1:100, SCB sc-33676). Then the sections were processed as previously described (40). Digital images were analyzed with ImageJ software (National Institutes of Health, Bethesda, MD, United States) using the color deconvolution plug-in. When the Immunohistochemistry Profiler plugin is selected, it mechanically plots a histogram profile of the deconvoluted diaminobenzidine image, and a corresponding scoring log is exhibited. The histogram profile refers to the positive pixel intensity value obtained from a computer program.

### 2.10 Preparation of bladder homogenate

Bladder tissue was collected and transferred to cold phosphate-buffered saline (pH 7.4). It was then cut into thin slices with a surgical scalpel, suspended in a cooled sucrose solution (0.25 M), and dried quickly on filter paper. Tissues were homogenized to release soluble proteins in a cooled tris hydrochloride buffer (10 mM, pH 7.4), and then centrifuged at 7000 rpm for 20 min as previously described (9).

### 2.11 Western blot

After harvesting, the spinal cord tissues of the L6-S1 segment were separated and immediately stored at −80°C for further analysis. Samples were lysed, proteins were separated by SDS-PAGE electrophoresis and subsequently transferred onto polyvinylidene fluoride membranes at 300 mA, as previously described (41). The membranes were then incubated overnight at 4°C with the following primary antibodies: c-Fos (E-8, 1:1000; sc-166940; SantaCruz Biotechnology), ionized calcium-binding adapter molecule 1 (Iba-1, 1:1000 v\v; ab5076, Abcam), phosphor-p38 (Tyr180/182, 1:1000; #4511, Cell Signaling Technology), glial fibrillary acidic protein (GFAP, 1:1000; #3670, Cell Signaling Technology), β-actin (1:1000; sc-47778; SantaCruz Biotecnology). Then, the membranes were washed three times and incubated with the secondary antibodies conjugated to horseradish peroxidase (1 h at 20–25°C). Protein bands were visualized using the Chemiluminescent ECL assay (Bio-Rad, Hercules, CA, United States) and visualized with the Chemi Doc XRS (Bio-Rad, Hercules, CA, United States). Band density was quantified using ImageJ analysis software (National Institutes of Health, United States).

### 2.12 Measurement of bladder histamine and serotonin levels

The expression levels of histamine and serotonin (5-hydroxytryptamine, 5-HT) in bladder tissue were measured with enzyme-linked immunosorbent assay (ELISA) kits (Elabscience Biotechnology Co., Ltd., Houston, Texas, 77,079, United States). The procedures were performed according to the manufacturer’s instructions, on bladder tissue homogenates obtained as described above. The results of histamine and 5-HT were expressed as pg./mL.

### 2.13 Real time-PCR

Total RNA was purified from bladder tissues using the RNeasy Kit according to the manufacturer’s protocol (Qiagen, Milan, Italy). RNA was then quantified using a Nanodrop Spectrometer. Reverse transcription of the extracted RNA was performed using RevertAid H Minus First Strand cDNA Synthesis Kit (ThermoFisher, Monza, Italy). cDNA samples were loaded in triplicate and were run according to the manufacturer’s settings on a BioRad CFX Connect RT-PCR machine using QuantiTect SYBR Green PCR Kits (Qiagen, Milan, Italy), and QuantiTech primers (Qiagen, Milan, Italy) according to specific manufacturer protocols. The 2^−ΔΔCq^ method was used for the calculation of the relative fold change, with gene expression being normalized to Glyceraldehyde 3-phosphate dehydrogenase (GAPDH).

### 2.14 Statistical analysis

All values are shown as mean ± standard error of the mean (SEM) of N observations. Figures are representatives of three independent experiments. The time-course data (i.e., von Frey testing) were analyzed by two-way ANOVA, while one-way ANOVA was used for all the other comparisons, with Kruskal-Wallis test being used for non-parametric variables. *Post hoc* evaluations were performed using Bonferroni adjustment for multiple comparisons. A value of p of less than 0.05 was considered significant. All analysis were performed on GrapPad Prism 10 software (GraphPad Software.USA).

## 3 Results

### 3.1 Effect of PGA-HSP on CYP-induced visceral pain

**Figure 2:**
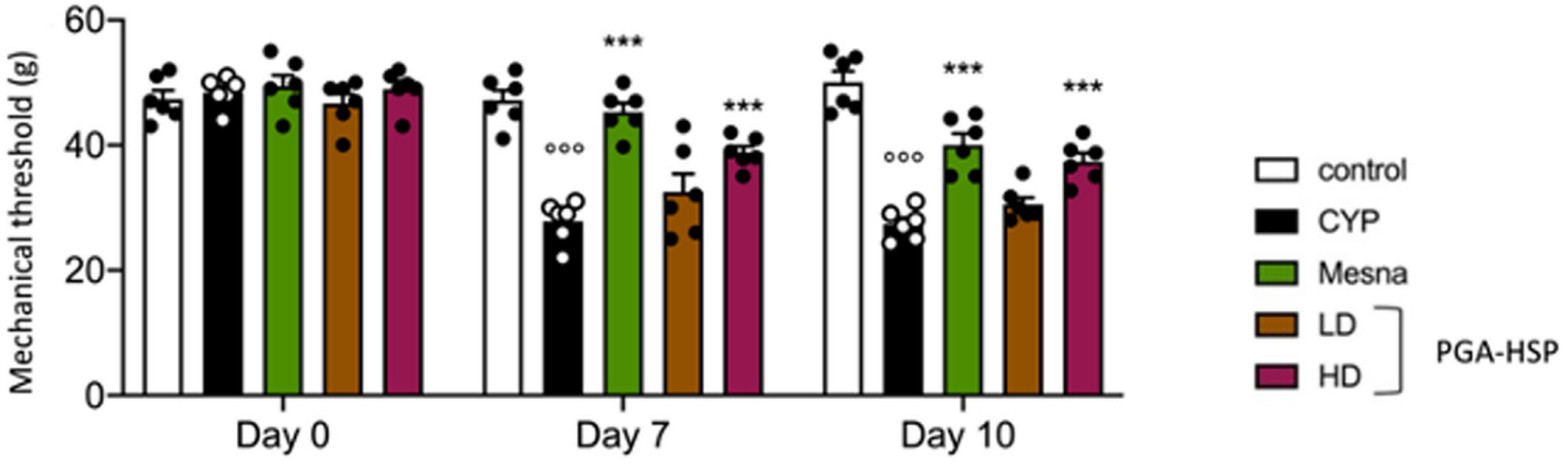
Von Frey test performed on the vicinity of the urinary bladder on baseline (day 0) and days 7 and 10 after three i.p. doses of CYP (40 mg/kg) or saline. Data are expressed as mean ± SEM (*n* = 6), figures are representative of three independent experiments. CYP, Cyclophosphamide; HD, high dose; LD, low dose; PGA-HSP, 3:1 mixture of micronized palmitoyl-glucosamine and hesperidin. ***p < 0.001 versus CYP; °°°*p* < 0.001 versus control.

The repeated injection of CYP produced persistent visceral pain as shown by the significant reduction in mechanical threshold compared to saline-injected controls, assessed by von Frey test at day 7 and day 10 (Figure 2). Not only mesna but also PGA-HSP (higher dose) relieved CYP-induced visceral pain at both timepoints, as shown by the significant increase of mechanical threshold compared to the CYP group (Figure 2).

### 3.2 Effect of PGA-HSP on CYP-induced changes in bladder wall thickness

**Figure 3:**
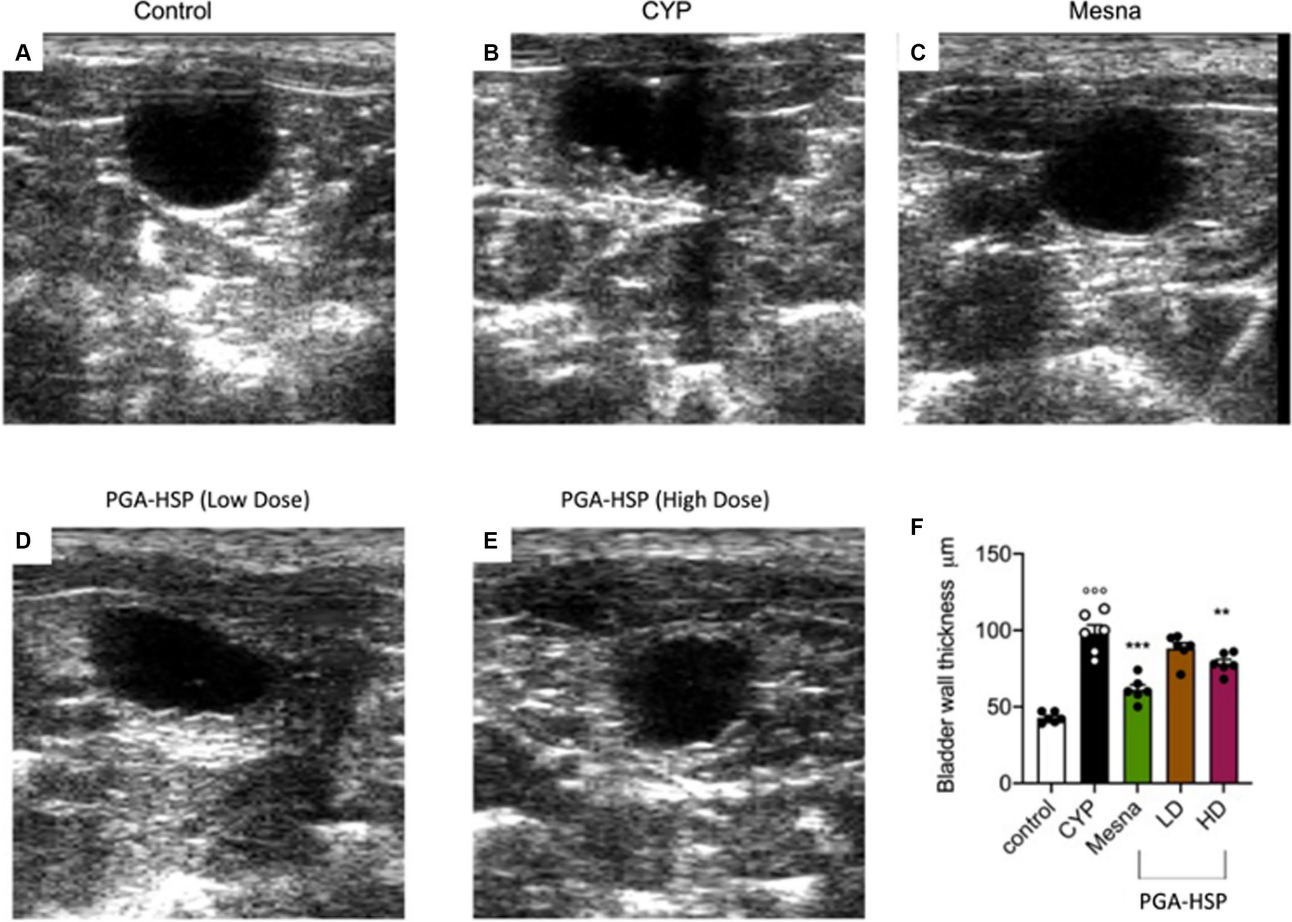
Ultrasound longitudinal view images of the urinary bladder in the different treatment groups, moderately distended with anechoic saline solution **(A–E)**. Bladder wall thickness in the different treatment groups is reported in the histogram **(F)**. Data are expressed as mean ± SEM (*n* = 6), figures are representative of three independent experiments. CYP, Cyclophosphamide; HD, high dose; LD, low dose; PGA-HSP, 3:1 mixture of micronized palmitoyl-glucosamine and hesperidin. **p < 0.01 versus CYP; ***p < 0.001 versus CYP; °°°*p* < 0.001 versus control.

At ultrasonography, a significant although irregular thickening of the bladder wall secondary to cystitis was observed in the longitudinal images from CYP-injected rats (Figure 3B) compared to controls (Figure 3A). The alteration was significantly reversed by both mesna (Figure 3C) and PGA-HSP at the higher dose (Figure 3E), as confirmed by the analysis of sonographic thickness of the bladder wall (Figure 3F).

### 3.3 Effect of PGA-HSP on CYP-induced damage to the urinary bladder: macroscopic observations

**Figure 4:**
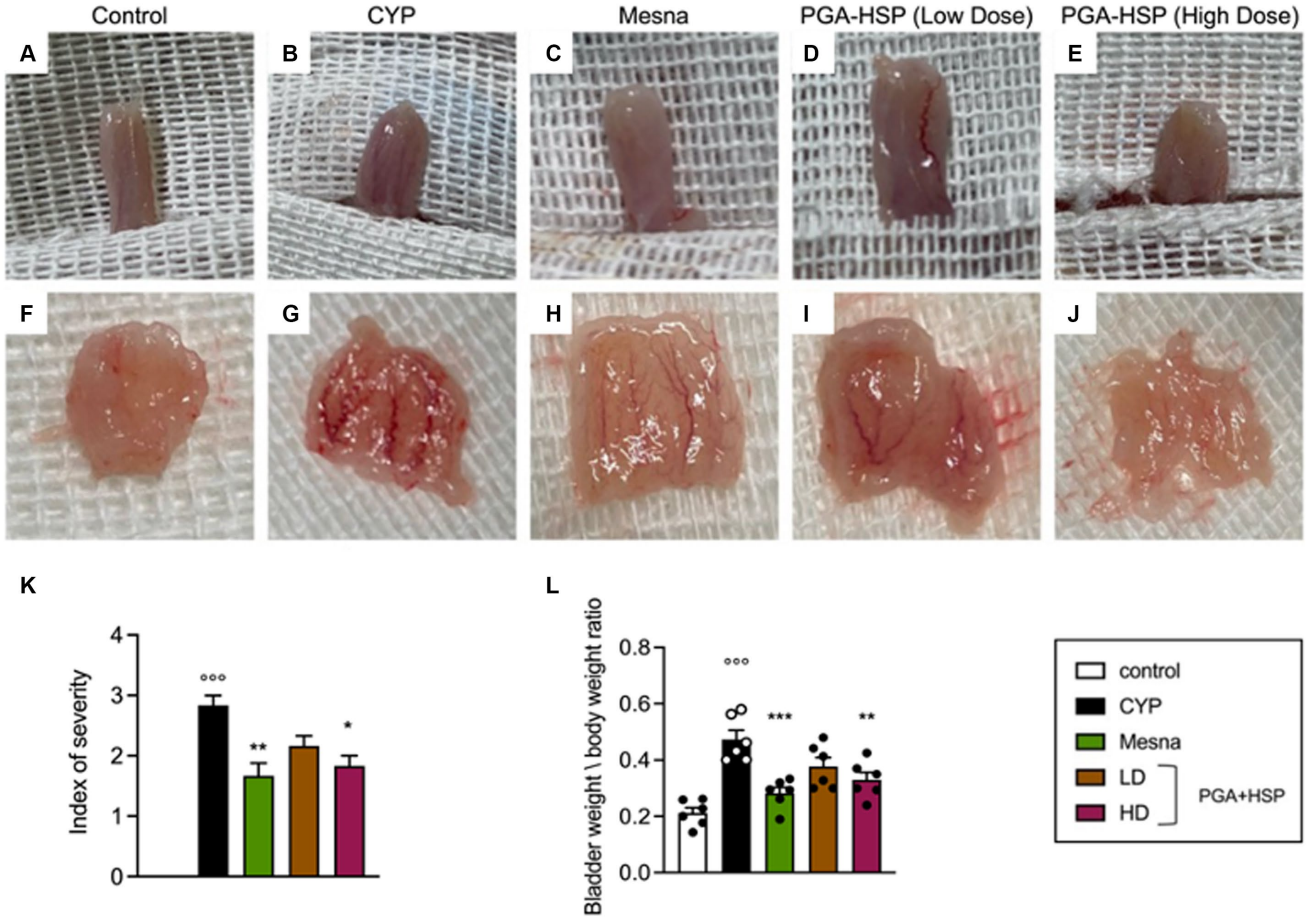
Macroscopic appearance of the whole urinary bladder **(A–E)** and its internal surface **(F–J)** from the different treatment groups. **(K)** Severity of cystitis-associated bladder damage according to the following scale (36): 3-severe (fluid in the bladder wall and internal mucosa, with evident bleeding), 2-moderate (fluid limited to the internal mucosa and little bleeding), 1-mild (little edema and no bleeding) and 0-normal bladder. **(L)** Bladder weight to body weight ratio reflecting mucosa edema. Data are expressed as mean ± SEM (*n* = 6), figures are representative of three independent experiments. CYP, Cyclophosphamide; HD, high dose; LD, low dose; PGA-HSP, 3:1 mixture of micronized palmitoyl-glucosamine and hesperidin. * *p* < 0.05 versus CYP, **p < 0.01 versus CYP; ***p < 0.001 versus CYP; °°°*p* < 0.001 versus control.

At the macroscopic examination, extensive edema and bleeding were observed in the urinary bladder of CYP-injected animals (Figures 4B,K), being consistent with severe cystitis (36, 42). Edema was observed both in the bladder wall and internal mucosa. The bladder-to-body weight ratio increased accordingly compared to control group (Figure 4L). At the higher dose, the study dietary intervention significantly reduced cystitis, approaching the efficacy of mesna, as shown both by the index of severity (Figure 4K) and bladder to body weight ratio (Figure 4L).

### 3.4 Effect of PGA-HSP on CYP-induced bladder inflammation, oxidative stress, and fibrosis

**Figure 5:**
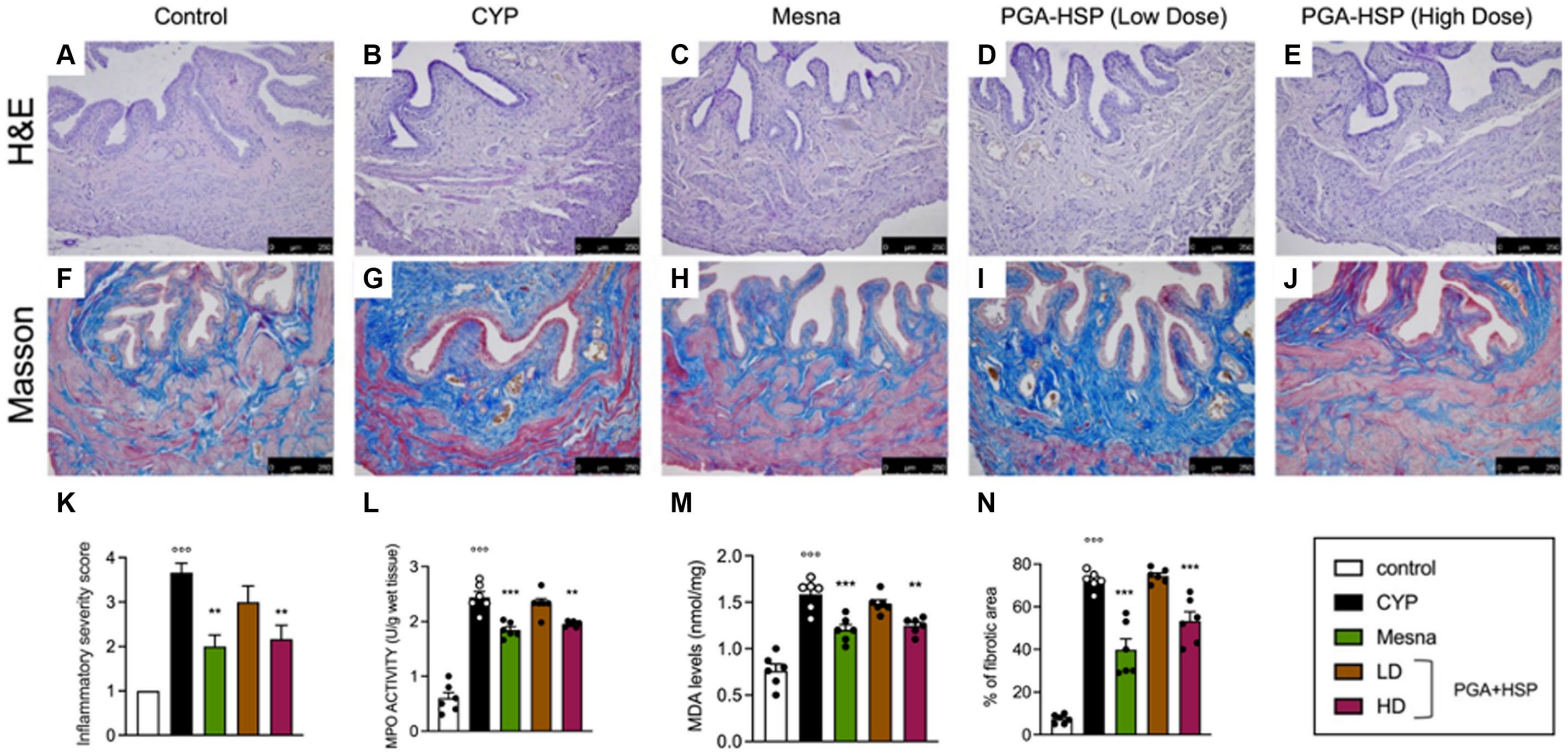
Representative photomicrographs of H&E staining of bladder tissue from the different experimental groups **(A–E)** and the relative inflammatory severity score **(K)**. MPO activity **(L)** and MDA levels **(M)** were quantified in bladder tissues from the different experimental groups. Masson’s trichrome staining images of bladders from the different experimental groups **(F–J)**, showing extensive increase of collagen deposition and hence fibrosis in the CYP group compared with controls (blue staining). The percentage of fibrotic area as quantified using ImageJ software is shown in the histogram **(N)**. Data are expressed as mean ± SEM (*n* = 6), figures are representative of three independent experiments. CYP, Cyclophosphamide; HD, high dose; LD, low dose; PGA-HSP, 3:1 mixture of micronized palmitoyl-glucosamine and hesperidin. **p < 0.01 versus CYP; ***p < 0.001 versus CYP; °°°*p* < 0.001 versus control.

Histological characterization showed that CYP injection resulted in inflammation, characterized by vascular congestion, microhemorrhages, and extensive submucosa edema (Figure 5B) compared to controls (Figure 5A). Accordingly, the inflammatory severity score significantly increased in CYP-injected animals compared to the control group (Figure 5K). Although the net activity level was still very low (i.e., lower than 3 units per gram of wet tissue), a significant increase of MPO activity was also observed following CYP injection (Figure 5L). The level of MDA also increased significantly compared to the control group (Figure 5M). Both the inflammatory and oxidative changes were similarly counteracted by mesna (Figures 5C,K–M) or the higher dose of the study supplement (Figures 5D,K–M). At Masson’s trichrome staining, increased collagen density along the deeper layers of the lamina propria and interstitial fibrosis between smooth muscle bundles were evident in the bladder of CYP-injected rats (Figure 5G) compared to controls (Figure 5F). Mucosal sloughing and denuded urothelial mucosa with a thinner layer of epithelial cells were also observed. Both mesna (Figure 5H) and the study supplement (higher dose, Figure 5J) reverted collagen deposition and significantly reduced fibrosis as shown by the percentage of fibrotic area compared to CYP group (Figure 5N).

### 3.5 Effect of PGA-HSP on the expression of inflammatory genes in the bladder

**Figure 6:**
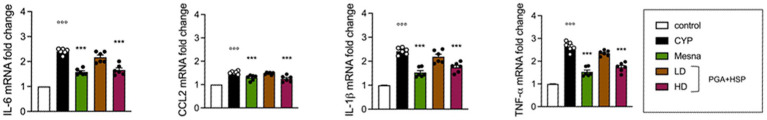
Bladder gene expression of IL-1β, TNF-α, IL-6 and CCL2 presented as mRNA fold change to control (set to 1). All values are expressed as mean ± SEM (*n* = 6). CYP, Cyclophosphamide; HD, high dose; LD, low dose; PGA-HSP, 3:1 mixture of micronized palmitoyl-glucosamine and hesperidin. ***p < 0.001 versus CYP; °°°*p* < 0.001 versus control.

The bladder mRNA expression level of the inflammatory cytokines IL-1β, TNF-α, IL-6 and CCL2 was significantly increased in the CYP group compared to saline-injected controls (Figure 6). PGA-HSP at the higher dose was able to significantly downregulate the gene expression of the investigated cytokines (Figure 6), and similar results were observed in the mesna-treated group.

### 3.6 Effect of PGA-HSP on bladder mast cells

**Figure 7:**
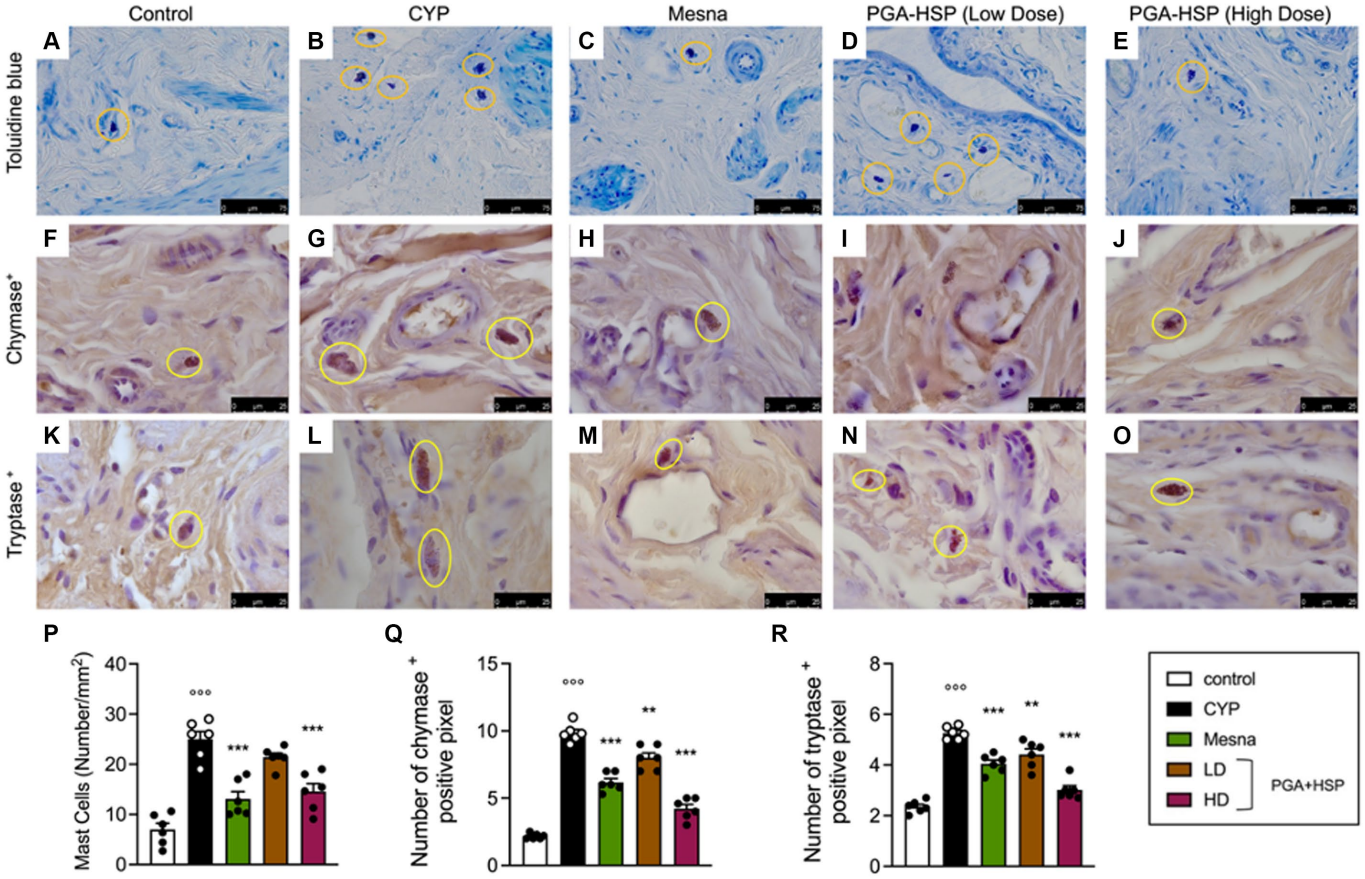
Bladder mucosal mast cells from the different treatment groups (yellow circles) in sections stained with toluidine blue **(A–E)** and immunostained for chymase **(F–J)** or tryptase **(K–O)**. The mast cell density (i.e., the number of mast cells per mm^2^) was calculated in 10 cross-sections at 40x magnification and shown in the histogram **(P)**. The number of chymase **(Q)** or tryptase positive pixels **(R)** was calculated with ImageJ software. Data are expressed as mean ± SEM (*n* = 6), figures are representative of three independent experiments. CYP, Cyclophosphamide; HD, high dose; LD, low dose; PGA-HSP, 3:1 mixture of micronized palmitoyl-glucosamine and hesperidin. **p < 0.01 versus CYP; ***p < 0.001 versus CYP; °°°*p* < 0.001 versus control.

Metachromatic staining of bladder mucosal mast cells showed a significant three-fold increase in cell density in CYP-injected rats compared to controls (Figures 7A,B,P). Similarly, significantly increased numbers of chymase- (Figures 7F,G,Q) and tryptase-positive mast cells (Figures 7K,L,R) were found in the bladder of CYP-injected animals compared to controls. Not only mesna but also the higher-dose PGA-HSP significantly decreased mucosal mast cell density at toluidine blue (Figures 7C,E,P) and immunohistochemical chymase (Figures 7H,J,Q) and tryptase staining (Figures 7M,O,R). Interestingly, the lower-dose PGA-HSP was also significantly effective in reducing the CYP-induced increase of both chymase- (Figures 7I,Q) and tryptase-positive mast cells (Figures 7N,R).

**Figure 8:**
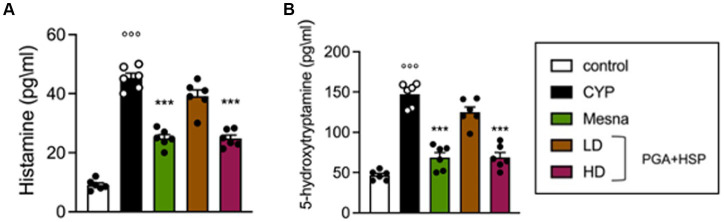
Histamine **(A)** and 5-HT levels **(B)** as determined by ELISA in bladder tissues. Data are expressed as mean ± SEM (*n* = 6), figures are representative of three independent experiments. CYP, Cyclophosphamide; HD, high dose; LD, low dose; PGA-HSP, 3:1 mixture of micronized palmitoyl-glucosamine and hesperidin. ***p < 0.001 versus CYP; °°°*p* < 0.001 versus control.

Since bladder mast cells are the main and candidate sources of histamine and 5-HT respectively, (43, 44), the level of the two mediators was evaluated in bladder tissues, as an index of degranulation (Figure 8). CYP significantly increased the level of both biogenic amines, while treatment with PGA-HSP (high dose) significantly counteracted the increase, with the effect being similar to that of mesna (Figures 8A,B).

### 3.7 Effect of PGA-HSP on CYP-induced alteration of the urothelial barrier

**Figure 9:**
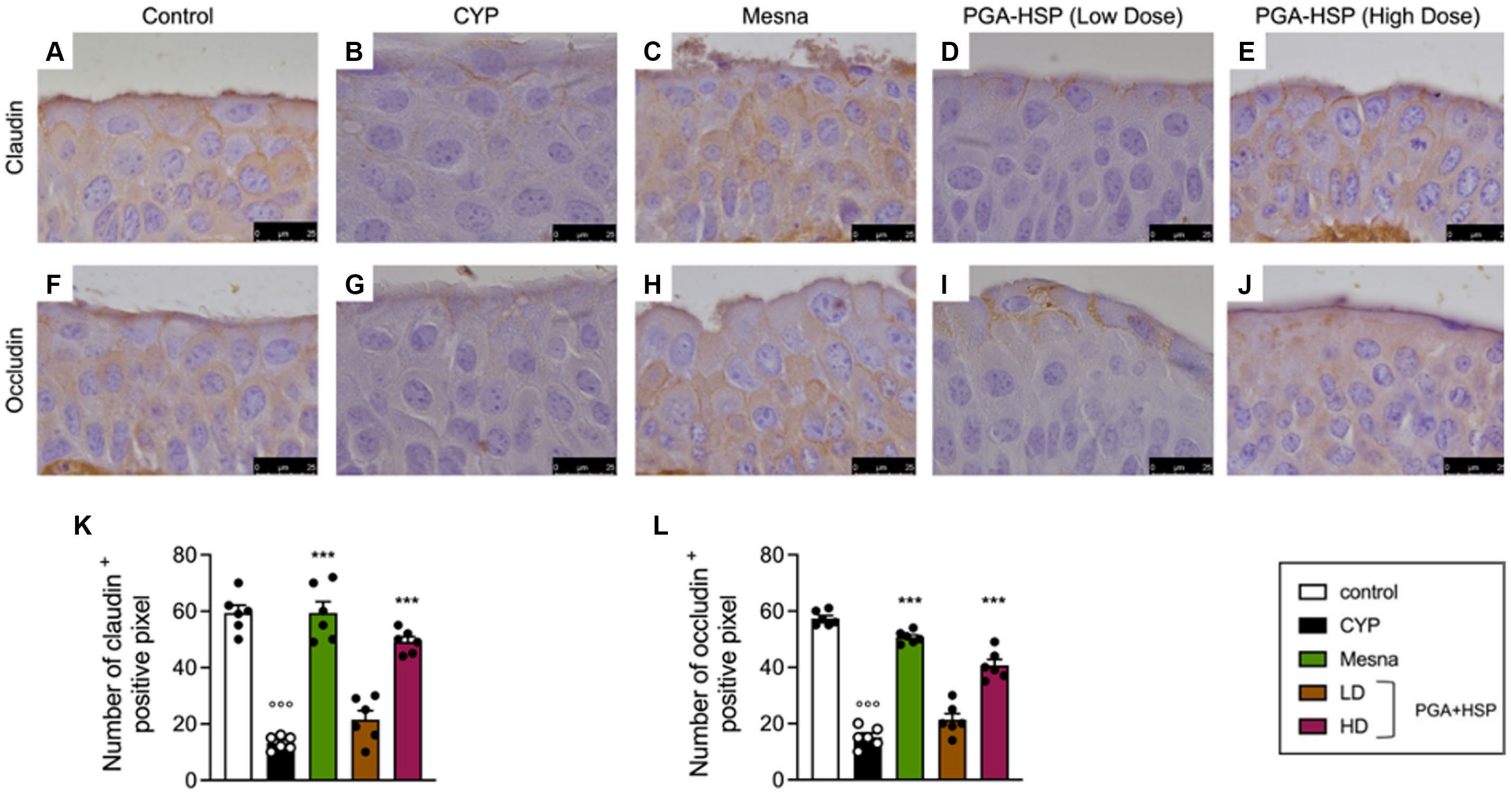
Immunohistochemical staining for claudin-1 **(A–E)** and occludin **(F–J)** in bladder tissue from the different treatment groups. The histograms **(K,L)** represent the number of positive pixels calculated with ImageJ software. Data are expressed as mean ± SEM (*n* = 6), figures are representative of three independent experiments. CYP, Cyclophosphamide; HD, high dose; LD, low dose; PGA-HSP, 3:1 mixture of micronized palmitoyl-glucosamine and hesperidin. ***p < 0.001 versus CYP; °°°*p* < 0.001 versus control.

In order to investigate whether the bladder epithelial barrier was compromised following CYP injection, the expression of the transmembrane barrier proteins claudin-1 and occludin was studied. Compared to the control group (Figures 9A,F), CYP-injected rats had reduced bladder expression of claudin-1 (Figures 9B,K) and occludin (Figures 9G,L), which was significantly restored by mesna (Figures 9C,H,K,L) as well as PGA-HSP at higher dose (Figures 9E,J–L).

### 3.8 Effect of PGA-HSP on CYP-induced activation of glial cells and neurons at the spinal level

**Figure 10:**
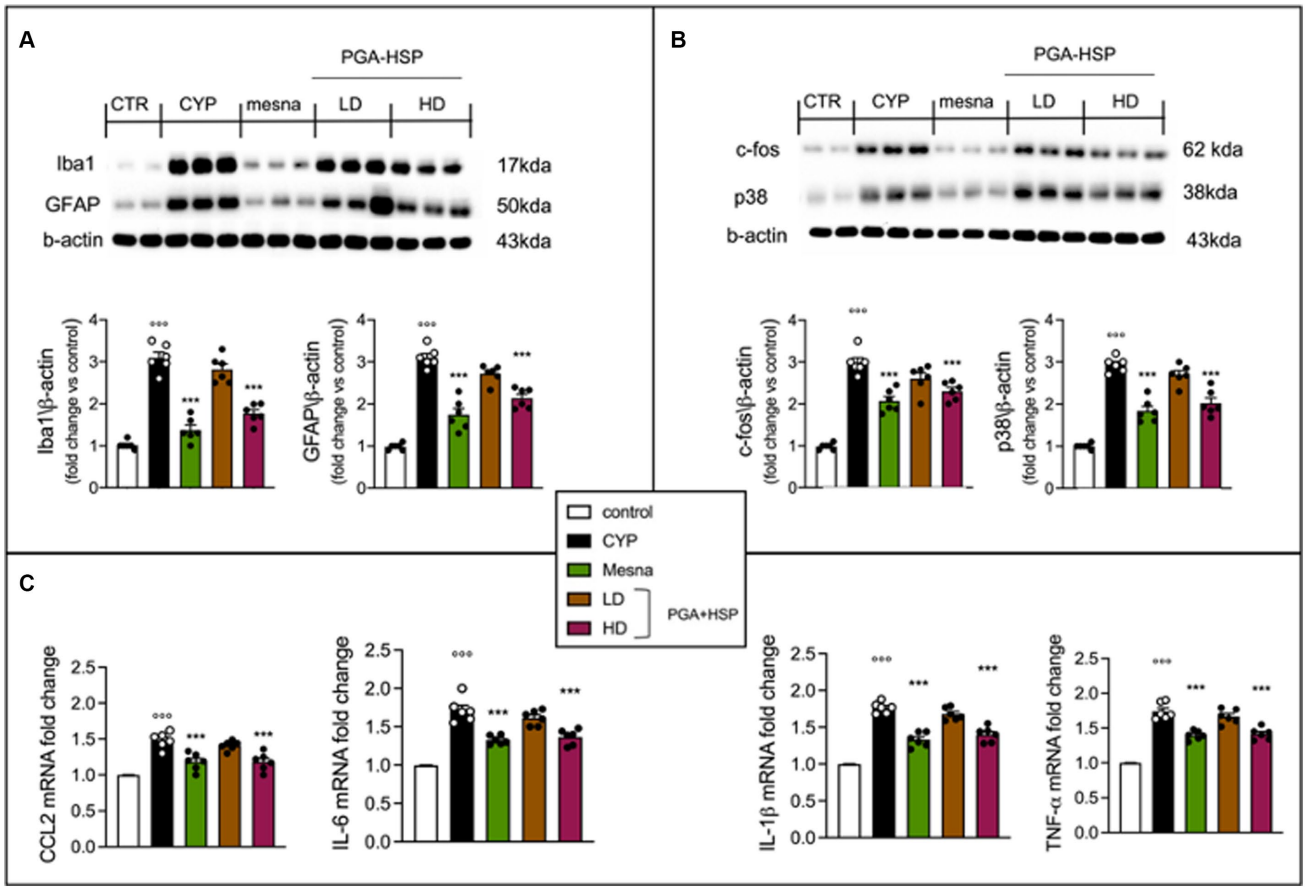
Western blot analysis of the activation markers Iba-1, GFAP **(A)**, c-fos and p38 **(B)**. Quantitative analysis of western blots was performed and levels were normalized relative to β-actin. Real time PCR assay for IL-1β, TNF-α, IL-6 and CCL2 **(C)**. All results are presented as fold change to control (set to 1). All values are expressed as mean ± SEM (*n* = 6). CYP, Cyclophosphamide; HD, high dose; LD, low dose; PGA-HSP, 3:1 mixture of micronized palmitoyl-glucosamine and hesperidin. ***p < 0.001 versus CYP; °°°*p* < 0.001 versus control.

Since accumulating evidence suggest that neuroinflammation plays an important role in the pathogenesis of cystitis (45, 46), we also evaluated the effect of PGA-HSP on CYP-induced neuroinflammatory changes in the lumbosacral spinal cord. Western blot analysis showed a significant increase of Iba-1 and GFAP expression (indicative of microglia and astrocyte activation, respectively) in the CYP group compared to controls (Figure 10A). Moreover, a significant increase of c-fos and p38 protein expression (activation markers for spinal neurons and microglia, respectively) was also observed in response to CYP (Figure 10B). Finally, the spinal mRNA expression level of the inflammatory cytokines IL-1β, TNF-α, IL-6 and CCL2 was also significantly increased in the CYP group compared to saline-injected controls (Figure 10C). Treatment with PGA-HSP at the higher dose exerted a significant protective effect on CYP-induced neuroinflammatory changes, with the mixture being able to significantly counteract the increase in all spinal cell activation markers (Figures 10A,B), as well as cytokine expression levels (Figure 10C), at a similar extent as the reference drug mesna.

### 3.9 Effect of PGA-HSP on spinal cord mast cells

**Figure 11:**
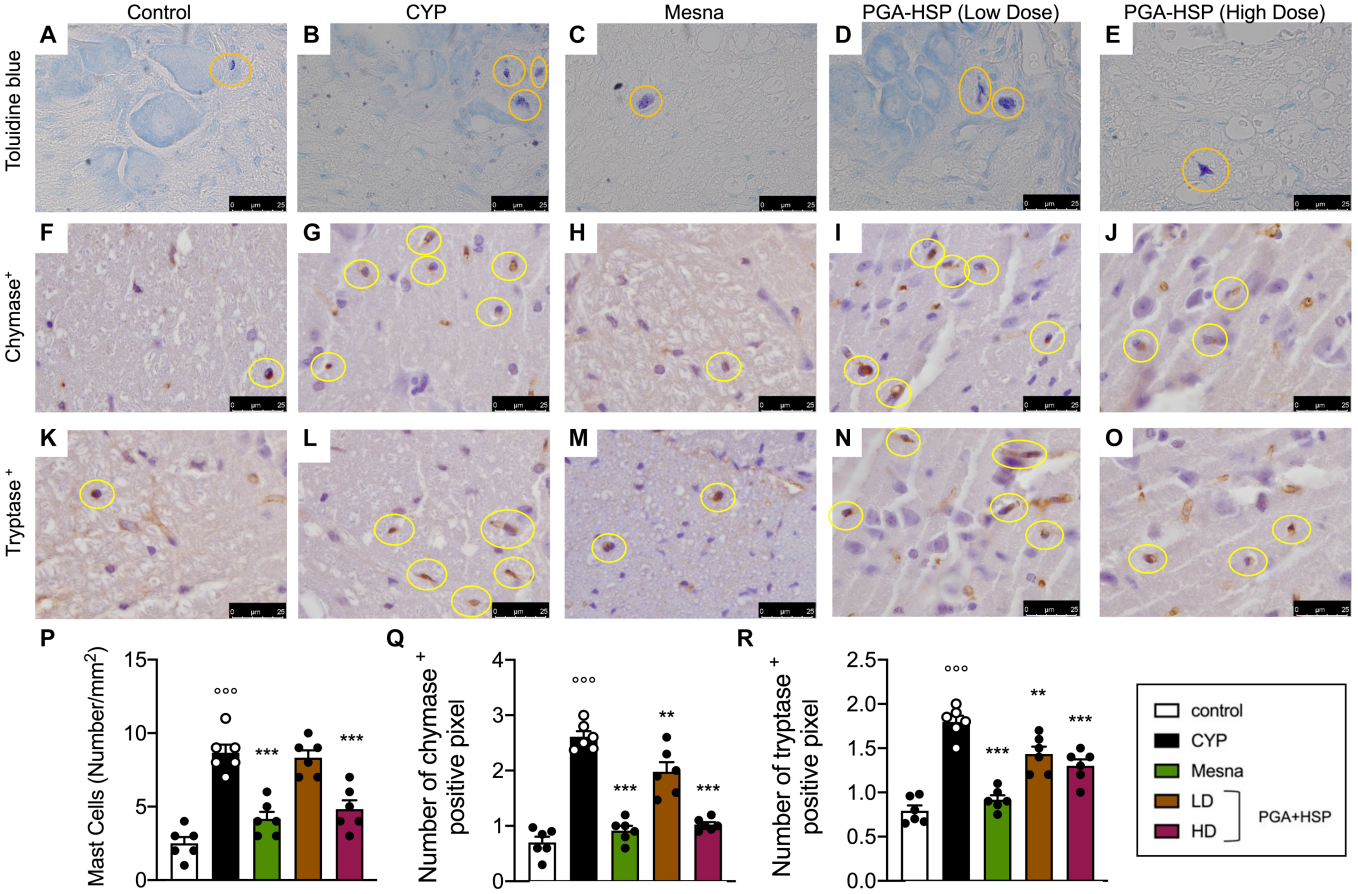
Mast cells in the spinal cord of the different treatment groups (yellow circles) in sections stained with toluidine blue **(A–E)** and immunostained for chymase **(F–J)** or tryptase **(K–O)**. The mast cell density (i.e., the number of mast cells per mm^2^) was calculated in 10 cross-sections at 40x magnification and shown in the histogram **(P)**. The number of chymase- **(Q)** or tryptase-positive pixels **(R)** was calculated with ImageJ software. Data are expressed as mean ± SEM (*n* = 6), figures are representative of three independent experiments. CYP, Cyclophosphamide; HD, high dose; LD, low dose; PGA-HSP, 3:1 mixture of micronized palmitoyl-glucosamine and hesperidin. **p < 0.05 versus CYP; ***p < 0.001 versus CYP; °°°*p* < 0.001 versus control.

Mast cells play a key role in pain processes both in peripheral tissues and central nervous system, including the spinal cord (47). We thus evaluated spinal mast cell count from the different treatment groups. As shown in Figure 11, the number of mast cells in the spinal cord from CYP-injected group (Figure 11B) increased compared to control group (Figure 11A). The mast cell density significantly increased accordingly (Figure 11P). The increase was significantly reduced by PGA-HSP (higher dose) as well as mesna (Figure 11P). The number of chymase-and tryptase-positive spinal mast cells increased accordingly in response to CYP (Figures 11G,L). Moreover, PGA-HSP supplementation, either at the higher or lower dose, significantly blocked CYP-induced increase in the number of both chymase-and tryptase-positive cells, with similar results being observed with mesna treatment (Figures 11Q,R).

## 4 Discussion

Idiopathic/interstitial cystitis, either in the human and feline patient, is a diagnosis of exclusion; as such it is frequently characterized by diagnostic delays, prolonged pain and suffering as well as detrimental effect on quality of life (3, 48). Notably, if left untreated, bladder fibrosis can progress, peripheral and central sensitization may occur and the disease may become more severe (49). Effective, long-lasting and safe interventions, able to properly target pathophysiological changes, may thus represent valuable tools within the multimodal approach to FIC. Here we have shown for the first time that a 3:1 mixture of micronized palmitoyl-glucosamine and the antioxidant flavonoid hesperidin (PGA-HSP) exerts beneficial effects in a rat model of CYP-induced chronic cystitis by reducing inflammation (both at bladder and spinal level, i.e., neuroinflammation), relieving pain and protecting the urothelial barrier.

In agreement with the original characterization of the CYP-induced chronic cystitis model (16), persistent visceral pain was observed in the present study following CYP injections. The pain-relieving effect of PGA-HSP, as measured by decreased mechanical allodynia, was similar to that of the reference drug (i.e., mesna) and consistent with previous results on micronized PGA, although in different disease models (26, 50). Of note, intravesical dimethyl sulfoxide (i.e., the only FDA-approved bladder installation for interstitial cystitis) failed to show any effect on CYP-induced chronic visceral pain in a previous study (16). Spinal neuroinflammation (i.e., the main driving force of chronic pain via central sensitization) was also observed in response to repeated CYP injections. The results agree with the recently observed spinal glial activation following CYP-induced cystitis (41, 51, 52). Notably, PGA-HSP significantly reduced glial cell as well as neuron hyper-reactivity, as shown by the decrease of the respective activation markers (i.e., Iba-1, p38, GFAP, and c-fos) and the reduced spinal expression of mRNA for genes encoding inflammatory cytokines (IL-1β, TNF-α, IL-6 and CCL2). Moreover, a significant increase in toluidine blue, as well as chymase-and tryptase-positive mast cell numbers was observed at the spinal cord level in response to CYP. Mast cells are located in the dura mater of the spinal cord at the cervical, thoracic, and lumbar regions and are responsible for sensitizing central nociceptive pathways at these levels, leading to hyperalgesia (53–55). Besides decreasing glial cell and neuron hyper-reactivity, PGA-HSP was also shown to significantly down-modulate spinal mast cell accumulation. Of note, the effect was statistically significant even at the lower dose. Probably the effects observed may be secondary to a reduction of damage at the level of the bladder mucosa resulting in a reduction of inflammation and the pathway related to it, but as shown in previous studies, palmitoyl-glucosamine also has a CNS system effect where it is able to reduce neuroinflammation. Anti-neuroinflammatory activity might thus account for the here observed pain-relieving effect of PGA-HSP, in accordance with the down-modulation of hyperactive immune-inflammatory cells assumed for the ALIA mechanism (56). Several lines of evidence point to the involvement of TLR4 in neuroinflammation and cystitis-associated chronic pain, as summarized below. An altered TLR4-mediated inflammatory response was demonstrated in patients with IC/BPS (57) and TLR4 activation was shown to play a critical role in bladder nociception (58). Accordingly, the TLR4/NF-κB signaling pathway is considered a useful therapeutic target for controlling neuroinflammation (59) and TLR4 receptor blockade was found to be protective against CYP-induced cystitis (60). Interestingly, PGA has recently shown to be a TLR4 antagonist (17, 27) and HSP inhibited the TLR4-NF-κB pathway (61), thus suggesting that the anti-neuroinflammatory effect here observed by supplementing PGA-HSP might be partially mediated through TLR4.

Palmitoyl-glucosamine is recognized as an analgesic substance at the level of the central nervous system due to its mechanism of action, but in our experimental model, the observed analgesic effects may be attributed to a combination of both central action effects and those involving the reduction of tissue damage observed in our study. In addition to the pain-relieving effect, PGA-HSP was here shown to significantly reverse CYP-induced local changes in the macroscopic and histopathological appearance of the bladder, as well as barrier function of the urothelial layer. Bladder wall thickening in response to CYP was significantly, albeit moderately, counteracted by the high dose supplementation with PGA-HSP. *In vivo* ultrasound is a noninvasive imaging tool that has only recently been used in preclinical models of cystitis, with a correlation between bladder wall thickness and cystitis severity being found (62, 63). The decrease of bladder wall thickness in response to PGA-HSP (and the reference drug mesna) may thus be viewed as an improvement in the bladder condition. Accordingly, the histologic index of severity and bladder to body weight ratio were also significantly reduced by PGA-HSP, similar to mesna. These effects are comparable to previous findings, obtained either with the intraperitoneal administration of a potent anti-inflammatory and antioxidant compound (i.e., Ambroxol, 30 mg/kg) or bladder instillation of a PGA congener (i.e., Adelmidrol, 2%) together with sodium hyaluronate (0.1%), in similar albeit not identical CYP-induced cystitis models (36, 64).

The triad inflammation-oxidative stress-fibrosis was effectively mitigated by PGA-HSP supplementation (high dose), as manifested by the reduction of inflammatory severity score, MPO activity, MDA levels and percentage of fibrotic area within the bladder tissue. In accordance with the original characterization of the chronic cystitis model here used (16), the inflammatory response was not severe. The MPO net activity, for instance, was much lower than reported in acute cystitis models (36), thus suggesting a scarce neutrophil granulocyte infiltration. All the same, PGA-HSP was found to reduce inflammatory changes, as mirrored by decreased bladder gene expression of inflammatory cytokines (i.e., of IL-1β, TNF-α, IL-6 and CCL2). Interestingly, the effect was consistent with that observed in response to the pharmacological blockade of the TLR4 receptor (60). Similarly, the local accumulation of mast cells in bladder tissues was responsive to PGA-HSP supplementation. Of note, the massive increase of chymase-and tryptase-positive cells was significantly reduced not only by the high but also by low dose of the study mixture, with the magnitude of the effect being similar to the reference drug, mesna. The findings are particularly relevant in light of the massive increase of mast cell density in the bladder wall in the course of idiopathic/interstitial cystitis (4, 65–67). Given the pathogenetic role played by these cells in lower urinary tract disorders (68, 69), the effect of PGA-HSP on bladder mast cell accumulation holds considerable implications for the management of cystitis. The CYP-induced increase in bladder mast cell releasability was also counteracted by the study mixture, as manifested by the significant reduction of histamine and 5-HT (i.e., serotonin) levels in bladder tissues. Indeed, mast cells are a major source for histamine and a possible source of 5-HT in urinary bladder (70), with both mediators being involved in bladder inflammation and hypersensitivity (44, 70–72). Targeting bladder mast cell accumulation and degranulation may thus represent at least one of the mechanisms responsible for the uroprotective effect of PGA-HSP. Furthermore, bladder mast cells are considered to be involved in epithelial barrier dysfunction (73) and indeed alteration of the urothelial barrier was observed in the present study. Notably, the CYP-induced decrease of tight junction proteins (i.e., occludin and claudin-1) was significantly restored by PGA-HSP. Tight junctions contribute to the multifaceted urothelial barrier, together with the apical membrane and the glycosaminoglycan mucus layer covering the urothelial surface (74). Accordingly, decreased expression of tight junctions is involved in barrier dysfunction and leaky urothelium, with the resulting sensitization of bladder afferents due to the diffusion of urinary solutes (5). Ultimately, the present findings on occludin and claudin-1 immunostaining showed that study mixture protected the urothelium against the CYP-induced urothelial barrier dysfunction. Such findings may have clinical implications not only for FIC patients, but also for chemotherapy-induced cystitis in dogs (75).

The main limitation of the present study is the lack of a PGA-or HSP-only treatment group. This makes it difficult to discriminate between the effect of the two compounds. For instance, some evidence on the anti-neuroinflammatory activity of HSP has been documented as extensively reviewed (76), but could not be confirmed here due to the lack of a HSP treatment group. When designing the study protocol, we first tried to limit the use of animals, in line with the principles of the 3Rs (replacement, reduction, and refinement) and second, we considered more worthwhile to compare the results with a reference compound (i.e., mesna) and between two different doses of the study mixture. Nevertheless, future research investigating the effects of the individual compounds on chronic visceral pain, bladder inflammation and barrier urothelial dysfunction is warranted. Moreover, the metabolic fate of PGA (i.e., the eventual release of glucosamine) deserves further investigations.

In conclusion, the present study has shown that a 3:1 mixture of micronized PGA and HSP significantly counteracted the multiple pathological changes occurring in CYP-induced chronic cystitis. The decrease of inflammatory mediators as well as cell hyper-activity at the bladder and spinal level, together with the protection of the urothelial barrier and pain-relieving effect, might be of clinical relevance to FIC patients.

## Data availability statement

The original contributions presented in the study are included in the article/supplementary material, further inquiries can be directed to the corresponding authors.

## Ethics statement

The animal study was approved by University of Messina board ethic committee. The study was conducted in accordance with the local legislation and institutional requirements.

## Author contributions

EG: Writing – original draft, Writing – review & editing, Methodology. GF: Writing – review & editing, Conceptualization. YM: Writing – review & editing, Conceptualization. AP: Writing – review & editing, Conceptualization. DI: Writing – review & editing, Conceptualization. MC: Methodology, Writing – review & editing. RS: Methodology, Writing – review & editing. RF: Methodology, Writing – original draft. RD’A: Methodology, Writing – review & editing. FM: Methodology, Writing – review & editing. RP: Investigation, Project administration, Writing – review & editing. SC: Investigation, Project administration, Writing – review & editing. RC: Project administration, Writing – review & editing.
